# Are Madagascar's obligate grazing-lawns ancient and evolved with endemic herbivores, or recently selected by introduced cattle?

**DOI:** 10.1098/rsbl.2022.0212

**Published:** 2022-09-14

**Authors:** Grant S. Joseph, Colleen L. Seymour

**Affiliations:** ^1^ Percy FitzPatrick Institute of African Ornithology, University of Cape Town, Rondebosch 7701, South Africa; ^2^ South African National Biodiversity Institute, Kirstenbosch, Claremont 7735, South Africa

Consensus is growing that Madagascar's largest ecoregion, the Malagasy Central Highlands (MCH), was a habitat mosaic (including forest, woodland, ericoid-scrubland and grasslands) at the period of human settlement of the island [1]. What is less certain is the identity of the grazer purported to have evolved through ‘millions of years of grazer and grass coevolution' to form the obligate grazing-lawns found on the MCH [2, p. 8]. Obligate C_4_-grazing lawns are functionally unique, forming when minimally grazed tall grass swards shift to facultative grazing-lawns (also with tall grasses), before ultimately transforming to short-stature grasses, tolerant of trampling, that spread laterally via rhizomes and stolons [3]. Critical to their formation and maintenance is regular grazing by C_4_-specialists with high muzzle-width to body-size ratios like African hippopotamus (*Hippopotamus amphibius*) and wildebeest (*Connochaetes taurinus*, with *ca* 95% C_4_-grass consumption), as opposed to less morphologically adapted, narrow-muzzled mixed-feeders (feeding on a combination of woody, succulent and grass species) like impala (*Aepyceros melampus*, with only *ca* 50% C_4_-grass consumption [3]).

Resolution of the debate is central to an emergent functional approach to conservation on this island (a global biodiversity hotspot), that supports processes to facilitate ancient ecological patterns, and avoids practices that do not [1]. Studies of carbon (C) isotopes can yield insights into animal diets, as grazers (consuming C_4_-grasses) and browsers (consuming C_3_ woody or crassulacean acid metabalism (CAM) species) have different ratios of ^13^C isotopes in their bone collagen: pure C_4_-grazer values exceed −9‰, whereas obligate C_3_-woody feeders measure below −21.5‰. Hansford & Turvey ([4], p. 1) make a valuable contribution using new analyses of C isotopes, concluding that most Malagasy megafauna fed primarily on C_3_ and/or CAM, providing evidence of ‘widespread browsing ecology'.

For specialist grazers, prime candidates have been thought to be the various species of hippopotamus that once inhabited Madagascar [2]. These species are estimated to have gone extinct *ca* 1200–1050 BP, at the time when agro-pastoralism led to intensive conversion of forest to grassland [5]. Hansford & Turvey's [4] analyses conclude that Malagasy hippopotamus species were predominantly browsers, not grazers. They find that the elephant bird (*Aepyornis hildebrandti*) obtained up to 48% of its diet from C_4_-grasses, interpreting this as evidence for a grazing guild among Madagascar's Holocene megaherbivores. Here, we offer an alternative interpretation.

With C_4_-grass representing under half of dietary intake (the remainder being CAM succulent browse and/or C_3_ material), *Aepyornis hildebrandti* groups with African mixed-feeding browser guilds (e.g. *Aepyceros melampus, Litocranius walleri* and *Madoqua guentheri*) that favour proximity to woodland/scrubland/thicket [6], and not with C_4_-grazing specialists tolerant of treeless grasslands, that exert the type of top-down control that forms obligate grazing-lawns, like *Connochaetes taurinus* [3] (figure 1).

**Figure 1.**
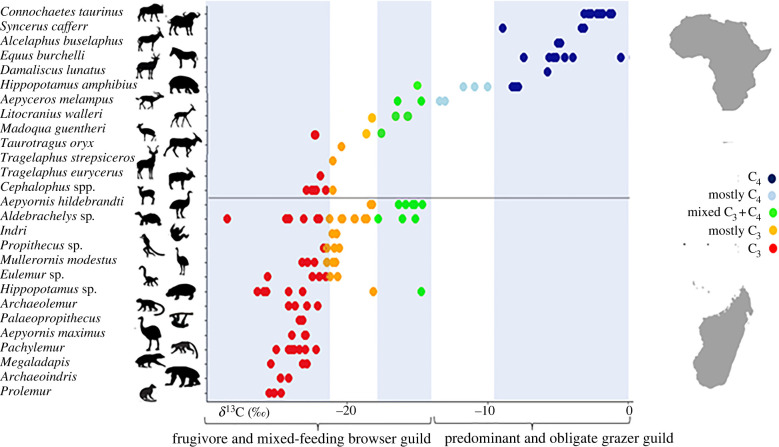
Suess-corrected δ^13^C collagen values for Malagasy subfossils, and modern African herbivores (for correction notes and data, see the electronic supplementary material, S1). No Malagasy grazing-guild ate mostly C_4_-grasses. African mixed-feeders regarded as browsers (e.g. *Aepyceros melampus*) have higher dietary C_4_-intake than any Malagasy subfossil species.

We also offer comment on comparisons with the diet of greater rhea (*Rhea americana*)*.* Rhea prefer legumes and other dicots, but also eat seeds, fruits, small vertebrates, invertebrates and tall grasses [7,8]. If *Aepyornis hildebrandti* had similar feeding habits, we would expect bill-feeding in minimally grazed swards that bear no functional similarity to cropped obligate grazing-lawn grasses (indeed, shorter pastoral-grasses negatively impact rhea [8]).

Importantly, endemic Malagasy grasses are sensitive to ungulate trampling and grazing, and probably ‘evolved under conditions of light grazing or no grazing' ([9], p. 6). Furthermore, Holocene isotope data support ‘significant tree cover from the last deglaciation', and ‘do not support widespread grasslands in central Madagascar' [10, p. 3]. Domestic cattle, introduced *ca* 1.5 ka, are the one species in Madagascar that can establish and maintain grazing-lawns [3], and evidence presented here supports MCH obligate grazing-lawns formed through top-down control by these broad-muzzled, C_4_-specialist grazers. Accepting that treeless grasslands were probably historically far smaller than today [11,12] averts an evolutionary anomaly: the absence of an indigenous C_4_-specialist grazer that has evolved with treeless grasslands (which today represent Madagascar's largest ecological niche). This, despite the presence of prime candidates, Malagasy hippopotamus species, which Hansford & Turvey [4] affirm were not specialist grazers.

## Data Availability

The data are provided in the electronic supplementary material [13].
